# Supplementary material for: The microbiome biomarkers of pregnant women’s vaginal area predict preterm prelabor rupture in Western China
Source: Front Cell Infect Microbiol. 2024 Oct 31;14:1471027. doi: 10.3389/fcimb.2024.1471027 (PMC11560878; doi:10.3389/fcimb.2024.1471027)
Supplement: Supplementary file 1 [file DataSheet1.zip › compare_1/Community/Pieplot/class/sample/P18.class.pieplot.pdf]

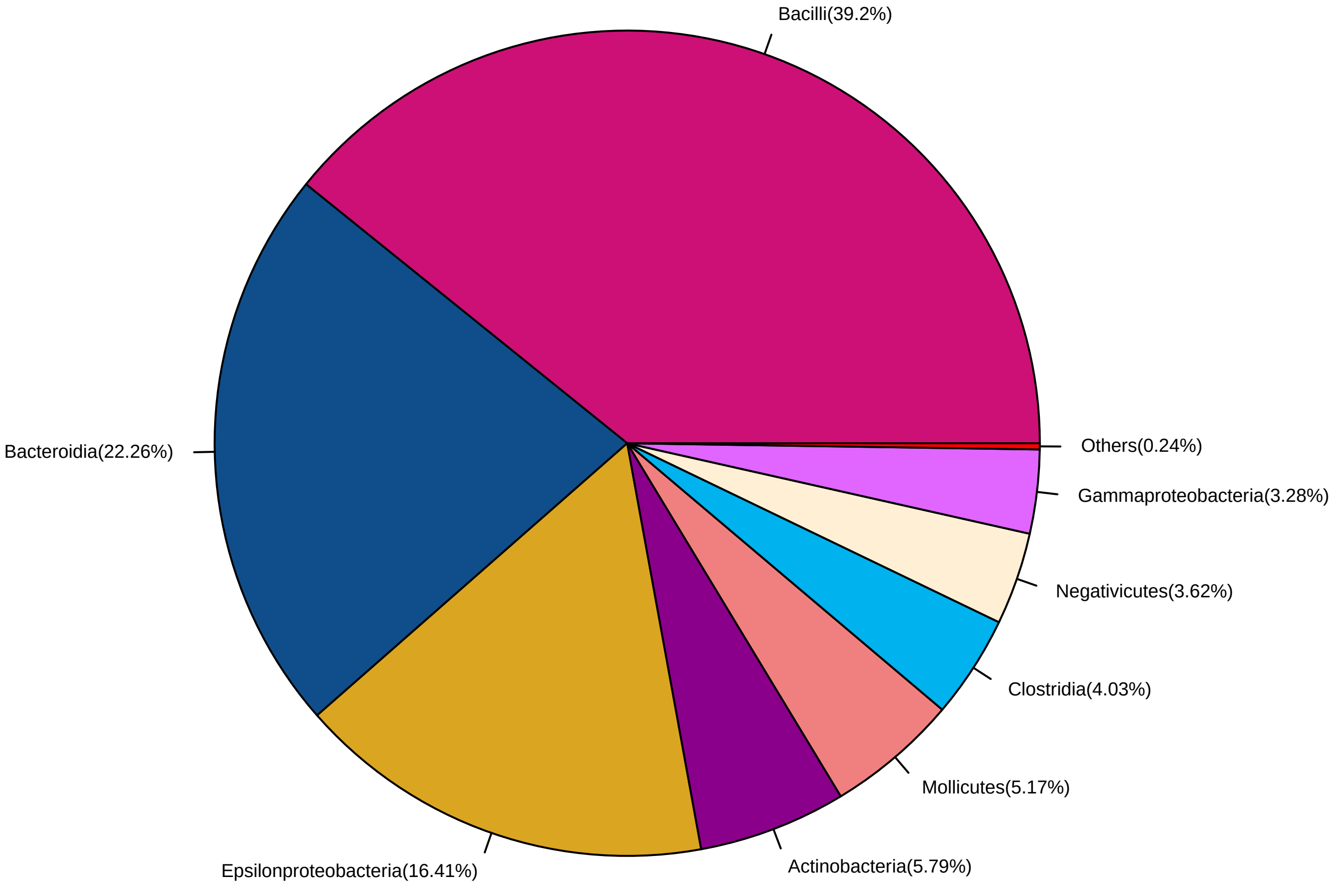
Bacilli(39.2%)
Others(0.24%)
Gammaproteobacteria(3.28%)
Negativicutes(3.62%)
Clostridia(4.03%)
Mollicutes(5.17%)
Actinobacteria(5.79%)
Epsilonproteobacteria(16.41%)
Bacteroidia(22.26%)
